# Overexpression of the IGF-II/M6P Receptor in Mouse Fibroblast Cell Lines Differentially Alters Expression Profiles of Genes Involved in Alzheimer’s Disease-Related Pathology

**DOI:** 10.1371/journal.pone.0098057

**Published:** 2014-05-20

**Authors:** Yanlin Wang, Gopal Thinakaran, Satyabrata Kar

**Affiliations:** 1 Department of Psychiatry, University of Alberta, Edmonton, Alberta, Canada; 2 Centre for Prions and Protein Folding Diseases, University of Alberta, Edmonton, Alberta, Canada; 3 Departments of Neurobiology, Neurology and Pathology, The University of Chicago, Chicago, Illinois, United States of America; 4 Department of Medicine (Neurology), University of Alberta, Edmonton, Alberta, Canada; Oregon Health & Science University, United States of America

## Abstract

Alzheimer’s disease (AD) is the most common type of senile dementia affecting elderly people. The processing of amyloid precursor protein (APP) leading to the generation of β-amyloid (Aβ) peptide contributes to neurodegeneration and development of AD pathology. The endocytic trafficking pathway, which comprises of the endosomes and lysosomes, acts as an important site for Aβ generation, and endocytic dysfunction has been linked to increased Aβ production and loss of neurons in AD brains. Since insulin-like growth factor-II (IGF-II) receptor plays a critical role in the transport of lysosomal enzymes from the trans-Golgi network to endosomes, it is likely that the receptor may have a role in regulating Aβ metabolism in AD pathology. However, very little is known on how altered levels of the IGF-II receptor can influence the expression/function of various molecules involved in AD pathology. To address this issue, we evaluated the expression profiles of 87 selected genes related to AD pathology in mouse fibroblast MS cells that are deficient in murine IGF-II receptor and corresponding MS9II cells overexpressing ∼500 times the human IGF-II receptors. Our results reveal that an elevation in IGF-II receptor levels alters the expression profiles of a number of genes including APP as well as enzymes regulating Aβ production, degradation and clearance mechanisms. Additionally, it influences the expression of various lysosomal enzymes and protein kinases that are involved in Aβ toxicity. IGF-II receptor overexpression also alters expression of several genes involved in intracellular signalling as well as cholesterol metabolism, which play a critical role in AD pathology. The altered gene profiles observed in this study closely match with the corresponding protein levels, with a few exceptions. These results, taken together, suggest that an elevation in IGF-II receptor levels can influence the expression profiles of transcripts as well as proteins that are involved in AD pathogenesis.

## Introduction

Alzheimer’s disease (AD), the most common type of senile dementia affecting elderly people, is characterized neuropathologically by extracellular β-amyloid (Aβ) peptide-containing neuritic plaques, intracellular tau-positive neurofibrillary tangles and the loss of neurons in selected regions of the brain. Although most AD cases occur sporadically after 65 years of age, a small proportion of cases correspond to the early-onset (<60 years) autosomal dominant form of the disease. To date, mutations in three genes - the β-amyloid precursor protein (*APP*) gene on chromosome 21, the presenilin 1 (*PSEN1*) gene on chromosome 14 and the presenilin 2 (*PSEN2*) gene on chromosome 1 - have been identified as the cause of a large proportion of early-onset familial AD. Additionally, inheritance of the ε4 allele of the apolipoprotein E (*APOE*) gene on chromosome 19 has been shown to increase the risk of late-onset and sporadic AD [1]–[5]. At present, the underlying cause for the AD pathogenesis remains unclear, but several lines of experimental evidence suggest that cerebral accumulation of Aβ peptides may initiate and/or contribute to the development of AD pathology [1], [6], [7]. These Aβ peptides are generated from their precursor APP, which is proteolytically processed by two alternative pathways; non-amyloidogenic α-secretase and amyloidogenic β-secretase pathways [8]–[11]. While the α-secretase pathway precludes the formation of intact Aβ peptides by cleaving APP within the Aβ domain, the β-secretase pathway yields the full-length Aβ_1–40_/Aβ_1–42_ peptides. The endosomal-lysosomal system, which comprises of tubulo-vesicular endocytic organelles and the lysosomes, has been shown to play a critical role in the production of Aβ peptides as well as to certain extent, intracellular degradation of nascent Aβ peptides. There is evidence that alternative processing of APP can be regulated by multiple factors that can influence not only the generation of Aβ but also the development/progression of AD pathology [10].

The insulin-like growth factor-II/mannose-6-phosphate (IGF-II/M6P or IGF-II) receptor, is a single transmembrane domain glycoprotein widely expressed in brain and peripheral tissues. The receptor binds two different classes of ligands: i) M6P-bearing molecules such as lysosomal enzymes, and ii) IGF-II - a mitogenic polypeptide with structural homology to IGF-I and insulin [12]–[14]. At the cellular level, a subset of the receptor is located at the plasma membrane, where it regulates internalization followed by activation/clearance of various ligands or activation of intracellular signalling cascades. The majority of the receptor, however, is present within the trans-Golgi network (TGN) and endosomal organelles where they transport newly synthesized lysosomal enzymes for subsequent delivery to lysosomes [15], [16]. Since the endosomal-lysosomal system plays a critical role in the generation of Aβ-related peptides [9]–[11], it is likely that the receptor may also be involved in regulating AD pathology. This is partly supported by the evidence that i) IGF-II receptor is expressed in a subset of Aβ-containing neuritic plaques and tau-positive neurofibrillary tangles in the AD brain [17] and ii) the receptor levels are altered in affected regions of the AD brain in individuals with *PSEN1* mutations or carrying *APOE* ε4 alleles [17], [18] and iii) the levels of the IGF-II receptor are increased along with lysosomal enzymes in mutant APP transgenic mice overproducing Aβ peptides [19]. Additionally, it has recently been shown that IGF-II receptor is a substrate for β-secretase [β-APP cleaving enzyme (BACE1)], which is involved in the generation of Aβ peptides from APP [20]. Notwithstanding these results, very little is known on how altered levels of the IGF-II receptor can influence the expression and/or function of various molecules involved in AD pathology. To address this issue, we evaluated, as a first step, the expression profiles of 87 selected genes associated AD pathology in well characterized mouse fibroblast MS cells that are deficient in murine IGF-II receptor and corresponding MS9II cells that overexpress the human IGF-II receptor [21], [22]. We use these cell lines as they have been studied extensively to characterize the role of IGF-II receptor on cell signalling and intracellular trafficking of lysosomal enzymes [22]–[25]. Additionally, no neuronal cell line that stably overexpresses IGF-II receptor is currently available. The alterations in gene expression profiles observed in MS9II cells vs MS cells were validated using Western blotting. Our results clearly show that IGF-II receptor overexpression enhances APP mRNA/protein levels and some of the enzymes involved in Aβ metabolism. Additionally, it influences the expression profiles of various lysosomal enzymes and molecules regulating Aβ toxicity as well as cholesterol metabolism that have been shown to be involved in AD pathology.

## Materials and Methods

### Materials

NuPAGE 4–12% Bis-Tris gels were purchased from Life technologies, Corp. (Burlington, ON, Canada). DNA isolation kit, RNeasy mini kit, SABiosciences’ RT^2^ First Strand Kit, RT^2^ SYBR Green/Fluorescein qPCR master mix and the 96-well Mouse Alzheimer’s disease RT^2^ Profile PCR Array were all from Qiagen Inc. (Mississauga, ON, Canada). The bicinchoninic acid (BCA) protein assay kit and enhanced chemiluminescence (ECL) kit were from ThermoFisher Scientific Inc. (Nepean, ON, Canada). Sources of all primary antibodies used in the study are listed in Table 1. All horseradish peroxidase (HRP)-conjugated secondary antibodies were purchased from Santa Cruz Biotechnology (Paso Robles, CA, USA). All other chemicals were from Sigma-Aldrich or Thermo Fisher Scientific.

**Table 1:** Details of the primary antibodies used in this study.

Antibody Type	Type	Immunogen	Dilution	Source
A disintegrin and metalloprotease 9 (ADAM9)	Polyclonal	H	1∶1000	EMD Millipore, Co.
Amyloid precursor protein (APP, clone 22C11)	Monoclonal	RC	1∶2000	Abcam
Anterior pharynx defective -1 (APH-1)	Polyclonal	S	1∶500	EMD Millipore, Co.
Apolipoprotein E (APOE)	Polyclonal	R	1∶1000	Gift from Dr. J.E. Vance
ATP-binding cassette, sub-family A, member 1 (ABCA1)	Polyclonal	H	1∶1000	Novus Biologicals, LLC
Cathepsin B	Polyclonal	H	1∶400	Santa Cruz Biotechnology, Inc.
Cathepsin D	Polyclonal	H	1∶200	Santa Cruz Biotechnology, Inc.
Cyclin-dependent kinase 5 (CDK5)	Polyclonal	H	1∶1000	Cell Signaling Technology
Glycogen synthase kinase (GSK) 3 beta	Monoclonal	H	1∶3000	Abcam
Insulin degrading enzyme (IDE)	Polyclonal	H	1∶1000	Abcam
Insulin-like growth factor-II (IGF-II)	Polyclonal	H	1∶500	Santa Cruz Biotechnology, Inc.
Insulin-like growth factor-II receptor	Polyclonal	H	1∶3000	Gift from Dr. Carolyn Scott
Low density lipoprotein receptor-related protein (LRP) 1	Polyclonal	H	1∶4000	Gift from Dr. J.E. Vance
Low density lipoprotein receptor-related protein (LRP) 6	Polyclonal	M	1∶1000	Gift from Dr. J.E. Vance
Presenilin 1 (PS1)	Monoclonal	H	1∶1000	EMD Millipore, Co.
Urokinase-type plasminogen activator (uPA)	Polyclonal	H	1∶200	Santa Cruz Biotechnology, Inc.
β-actin	Monoclonal	S	1∶5000	Sigma-Aldrich, Inc.
β-glucuronidase	Polyclonal	RC	1∶500	Novus Biologicals
β-site APP cleaving enzyme 1 (BACE1)	Monoclonal	H	1∶2000	R&D Systems

M: mouse peptide; H: human peptide; R: rat peptide; RC: recombinant peptide; S: synthetic peptide.

### Cell Culture

IGF-II receptor deficient mouse fibroblasts MS and corresponding MS cells stably transfected with human IGF-II receptor known as MS9II cells (originally developed by Dr. W.S. Sly, Saint Louis University, MO, USA) [21], [22] used in this study were obtained as generous gifts from Dr. R. C. Bleackley (University of Alberta, AB, Canada). The cells were maintained in Dulbecco’s modified Eagle’s medium supplemented with 0.1 g/L sodium pyruvate, 2.2 g/L sodium bicarbonate, Pen/Strep 25U, 3.2 mM methotrexate and 5% dialyzed fetal bovine serum. The culture media did not contain any IGF-II or IGF-I, but the ingredients of dialyzed fetal bovine serum remain unknown. MS and MS9II cells between passages 5 and 14 were used in all of our experiments. Cells were seeded at 1×10^4^ cells/cm^2^ and the medium was replaced every 3–4 days as described earlier [24]. Cultured MS and MS9II cells were harvested under basal conditions at 90% confluency as per the requirement of the specific protocol or stored at −80°C until further processing.

### RNA Extraction for PCR Array

Total RNA was isolated from MS and MS9II cells using RNeasy mini kit following manufacturer’s instructions and stored at −80°C. RNA concentrations were determined using a Nanodrop 1000 spectrophotometer (Thermo Fisher Scientific) and 260/230 nm and 260/280 nm absorbance ratio were analyzed to determine RNA purity.

### Real-time RT-PCR Array

At first 1 µg of total RNA was treated with genomic DNA elimination buffer at 42°C for 5 min to remove possible genomic DNA contamination. Following the elimination step, reverse transcription was carried out using the real-time RT-PCR First Strand Kit in accordance with the manufacturer’s protocol (SuperArray Biosciences Corp., MD). The resulting complementary DNA (cDNA) was diluted and combined with real-time RT-PCR SYBR Green/Fluorescein qPCR master mix and loaded onto a Mouse Alzheimer’s Disease RT^2^ Profiler PCR Array designed to profile the expression of 87 genes representative of biological pathways involved in APP/Aβ metabolism, cell signalling, intracellular trafficking cholesterol metabolism and cell death. All real-time PCR reactions were performed in a final volume of 25 µL using a MyiQ Real-Time PCR Detection System (Bio-Rad Laboratories, Inc., Canada) using a two-step cycling program: 10 min at 95°C (one cycle), 15 s at 95°C, followed by 1 min at 60°C (40 cycles). Data collection was performed during the annealing step (58°C) of each cycle and data were PCR-baseline subtracted and curve fitted. Threshold cycle (Ct) values were calculated using the instrument’s MyiQ optical software (Bio-Rad Laboratories, Inc.).

### PCR Data Normalization and Analysis

The data were analyzed using the SABiosciences PCR Array Data analysis software based on the comparative Ct method and expressed as relative fold differences in MS9II cells compared to MS cells as described earlier [26]. All Ct values ≥35 were considered a negative call. Quality control tests for PCR reproducibility, reverse transcription efficiency and level of genomic DNA contamination were included in each plate and monitored as per the supplier’s instructions. The expression levels of two housekeeping genes included in the PCR array: *Gapdh* and *Actb* were used for normalization. The ΔCt for each gene in each plate was first calculated by subtracting the Ct value of the gene of interest from the average Ct value of the two housekeeping genes. Then, the average ΔCt value of each gene was calculated across the four replicate arrays for each cell line and ΔΔCt values were obtained by subtracting the ΔCt values of MS cells from the respective ΔCt values of MS9II cells. The fold-change for each gene from MS cells to MS9II cells was calculated as 2∧ (−ΔΔCt). Finally the fold-change for each gene was converted to fold-regulation as follows. For fold-change values >1, which indicated a positive or an up-regulation, the fold-regulation was equal to the fold-change. For fold-change values <1 indicating a negative or down-regulation, the fold-regulation was calculated as the negative inverse of the fold-change. *P*-values were calculated using Student’s *t*-test. A fold difference of ≥1.2 with a *p*-value <0.05 was considered as significant differential gene expression.

### Immunocytochemistry

MS and MS9II cells seeded at 1×10^4^ cells/cm^2^ on coverslips were fixed with 4% paraformaldehyde for 15 min, washed with phosphate-buffered saline (PBS) and then incubated overnight at 4 C with anti-IGF-II receptor antibody. The coverslips were then exposed to appropriate Alexa Fluor 594-conjugated secondary antibodies (1∶1000) for 2 h. The cell nucleus was stained with 1 µg/mL Hoechst 33258 for 5 min. The coverslips were washed with PBS and mounted with ProLong Gold antifade medium as described earlier [27], [28]. Immunostained cells were visualized using a Zeiss LSM 510 confocal microscope and the images were analyzed with ZEN 2010 (Carl Zeiss, Germany).

### Western Blotting

Western blotting on cultured cell lysates was performed as described earlier [27], [28]. In brief, cultured cells were homogenized with radioimmunoprecipitation lysis buffer containing protease inhibitor cocktail and then proteins were quantified using a BCA kit. Denatured samples were resolved on 7–17% gradient sodium polyacrylamide or NuPAGE 4–12% Bis-Tris gels, transferred to polyvinylidene fluoride membranes, blocked with 5% skim milk and then incubated overnight at 4°C with different primary antibodies at proper dilutions as indicated in the Table 1. On the following day, membranes were incubated with appropriate HRP-conjugated secondary antibodies (1∶5000) and immunoreactive proteins were visualized using an ECL detection kit. All blots were re-probed with anti-β-actin antibody and quantified using a Microcomputer Imaging Device (MCID) image analysis system (Imaging Research, Inc., St Catherines, ON, Canada) as described earlier [27], [28].

### Statistical Analysis

All data expressed as means ± SEM were obtained from four separate batches of cultures. Comparisons between two groups were performed using Student’s *t*-test. A *p* value of less than 0.05 was accepted as statistically significant. All statistical analyses were performed using GraphPad Prism (GraphPad software, Inc., CA, USA).

## Results

### Real-time RT-PCR Array Analysis of Gene Expression

**Figure 1:**
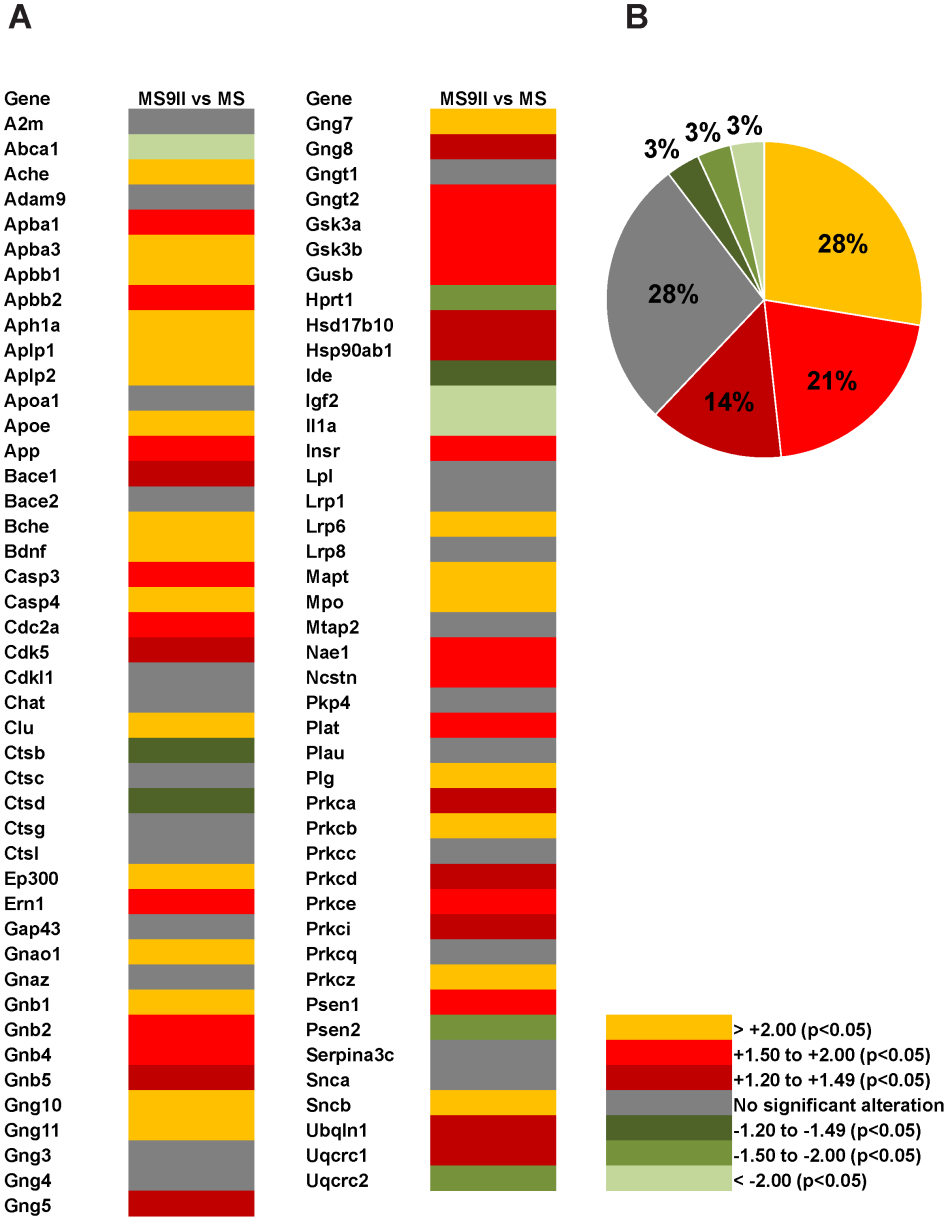
Heat-map diagram showing gene expression profiles in MS and MS9II cells. The figure represents data obtained using mouse AD-PCR-Array of 87 selected genes involved in APP/Aβ metabolism, cholesterol metabolism, lysosomal enzyme and cell signalling. Transcriptional levels are colored yellow and different shades of red for significant up-regulations, different shades of green for significant down-regulations and grey for no alteration in MS9II cells compared to MS cells. Of the 87 genes evaluated, 54 transcripts are up-regulated and 9 genes are down-regulated, while remaining 24 genes remained unaltered in MS9II cells compared to MS cells (A). Pie-chart showing percentage of up- and down-regulated genes in MS9II cells compared to MS cells. Gene expression levels are colored yellow and shades of red for significant up-regulation, various shades of green for significant down-regulations and grey for no alteration. As evident from the pie-charts, several genes are differentially altered following overexpression of the human IGF-II receptor in MS9II cells (B). The data included in the heat-map diagram were obtained from four different experiments.

In order to gain molecular insights on the influence of the IGF-II receptor overexpression on AD pathology, we used well-characterized IGF-II receptor deficient MS cells and the corresponding MS9II cells that stably overexpresses the human IGF-II receptor ∼500 times compared to MS cells [21], [22]. These cells have been used extensively not only to define the role of the receptor in intracellular trafficking of the lysosomal enzymes but also in establishing its implication in cell signalling [23]–[25]. In our study we analyzed the expression profiles of 87 selected genes (Table 2) involved in APP/Aβ metabolism, cell signalling, cholesterol metabolism and cell death mechanism in MS and MS9II cells using real-time RT-PCR array. Our results revealed marked alterations in the relative expression of a wide-spectrum of transcripts in MS9II cells compared to MS cells (Fig. 1A). Complete list of differentially regulated genes with the respective fold-change in MS9II cells *vs* MS cells are provided in Table S1. Of the 87 genes evaluated, 54 genes (e.g. *App, Aph1a, Apoe, Aplp1, Aplp2, Bace1, Cdk5, Clu, Gsk3a, Gsk3b, Gusb, Psen1 and Ncstn etc.*) were significantly (*p*<0.05) up-regulated and 9 genes (e.g., *Abca1, Ctsb, Ctsd, Igf2 and Ide*) were significantly (*p*<0.05) down-regulated, while remaining 24 genes (e.g., *A2m*, *Adam9*, *Bace2, Gap43, Ctsg, Ctsl* and *Plau*) were unaltered in MS9II cells compared to MS cells (Fig. 1B). The majority of the differentially expressed genes showed 1.2- to 2-fold changes, whereas only few genes such as *Apoe*, *Aph1a*, *Aplp1, Aplp2, Clu, Igf2* and *Abca1* displayed more than 2-fold changes.

**Table 2:** List of selected genes for Mouse Alzheimer’s disease real-time RT-PCR array.

NCBI Ref Seq#	Gene Symbol	Official Gene Name
NM_175628	*A2m*	Alpha-2-macroglobulin
NM_013454	*Abca1*	ATP-binding cassette, sub-family A (ABC1), member 1
NM_009599	*Ache*	Acetylcholinesterase
NM_007404	*Adam9*	A disintegrin and metallopeptidase domain 9 (meltrin gamma)
NM_177034	*Apba1*	Amyloid beta (A4) precursor protein binding, family A, member 1
NM_018758	*Apba3*	Amyloid beta (A4) precursor protein-binding, family A, member 3
NM_009685	*Apbb1*	Amyloid beta (A4) precursor protein-binding, family B, member 1
NM_009686	*Apbb2*	Amyloid beta (A4) precursor protein-binding, family B, member 2
NM_146104	*Aph1a*	Anterior pharynx defective 1a homolog (C. elegans)
NM_007467	*Aplp1*	Amyloid beta (A4) precursor-like protein 1
NM_009691	*Aplp2*	Amyloid beta (A4) precursor-like protein 2
NM_009692	*Apoa1*	Apolipoprotein A-I
NM_009696	*Apoe*	Apolipoprotein E
NM_007471	*App*	Amyloid beta (A4) precursor protein
NM_011792	*Bace1*	Beta-site APP cleaving enzyme 1
NM_019517	*Bace2*	Beta-site APP-cleaving enzyme 2
NM_009738	*Bche*	Butyrylcholinesterase
NM_007540	*Bdnf*	Brain derived neurotrophic factor
NM_009810	*Casp3*	Caspase 3
NM_007609	*Casp4*	Caspase 4, apoptosis-related cysteine peptidase
NM_007659	*Cdc2a*	Cell division cycle 2 homolog A (S. pombe)
NM_007668	*Cdk5*	Cyclin-dependent kinase 5
NM_183294	*Cdkl1*	Cyclin-dependent kinase-like 1 (CDC2-related kinase)
NM_009891	*Chat*	Choline acetyltransferase
NM_013492	*Clu*	Clusterin
NM_007798	*Ctsb*	Cathepsin B
NM_009982	*Ctsc*	Cathepsin C
NM_009983	*Ctsd*	Cathepsin D
NM_007800	*Ctsg*	Cathepsin G
NM_009984	*Ctsl*	Cathepsin L
NM_177821	*Ep300*	E1A binding protein p300
NM_023913	*Ern1*	Endoplasmic reticulum (ER) to nucleus signalling 1
NM_008083	*Gap43*	Growth associated protein 43
NM_010308	*Gnao1*	Guanine nucleotide binding protein, alpha o
NM_010311	*Gnaz*	Guanine nucleotide binding protein, alpha z subunit
NM_008142	*Gnb1*	Guanine nucleotide binding protein (G protein), beta 1
NM_010312	*Gnb2*	Guanine nucleotide binding protein (G protein), beta 2
NM_013531	*Gnb4*	Guanine nucleotide binding protein (G protein), beta 4
NM_010313	*Gnb5*	Guanine nucleotide binding protein (G protein), beta 5
NM_025277	*Gng10*	Guanine nucleotide binding protein (G protein), gamma 10
NM_025331	*Gng11*	Guanine nucleotide binding protein (G protein), gamma 11
NM_010316	*Gng3*	Guanine nucleotide binding protein (G protein), gamma 3
NM_010317	*Gng4*	Guanine nucleotide binding protein (G protein), gamma 4
NM_010318	*Gng5*	Guanine nucleotide binding protein (G protein), gamma 5
NM_010319	*Gng7*	Guanine nucleotide binding protein (G protein), gamma 7
NM_010320	*Gng8*	Guanine nucleotide binding protein (G protein), gamma 8
NM_010314	*Gngt1*	Guanine nucleotide binding protein (G protein), gamma transducing activity polypeptide 1
NM_023121	*Gngt2*	Guanine nucleotide binding protein (G protein), gamma transducing activity polypeptide 2
NM_001031667	*Gsk3a*	Glycogen synthase kinase 3 alpha
NM_019827	*Gsk3b*	Glycogen synthase kinase 3 beta
NM_010368	*Gusb*	Glucuronidase, beta
NM_016763	*Hsd17b10*	Hydroxysteroid (17-beta) dehydrogenase 10
NM_031156	*Ide*	Insulin degrading enzyme
NM_010514	*Igf2*	Insulin-like growth factor 2
NM_010554	*Il1a*	Interleukin 1 alpha
NM_010568	*Insr*	Insulin receptor
NM_008509	*Lpl*	Lipoprotein lipase
NM_008512	*Lrp1*	Low density lipoprotein receptor-related protein 1
NM_008514	*Lrp6*	Low density lipoprotein receptor-related protein 6
NM_053073	*Lrp8*	Low density lipoprotein receptor-related protein 8, apolipoprotein e receptor
NM_010838	*Mapt*	Microtubule-associated protein tau
NM_010824	*Mpo*	Myeloperoxidase
NM_001039934	*Mtap2*	Microtubule-associated protein 2
NM_144931	*Nae1*	NEDD8 activating enzyme E1 subunit 1
NM_021607	*Ncstn*	Nicastrin
NM_026361	*Pkp4*	Plakophilin 4
NM_008872	*Plat*	Plasminogen activator, tissue
NM_008873	*Plau*	Plasminogen activator, urokinase
NM_008877	*Plg*	Plasminogen
NM_011101	*Prkca*	Protein kinase C, alpha
NM_008855	*Prkcb*	Protein kinase C, beta
NM_011102	*Prkcc*	Protein kinase C, gamma
NM_011103	*Prkcd*	Protein kinase C, delta
NM_011104	*Prkce*	Protein kinase C, epsilon
NM_008857	*Prkci*	Protein kinase C, iota
NM_008859	*Prkcq*	Protein kinase C, theta
NM_008860	*Prkcz*	Protein kinase C, zeta
NM_008943	*Psen1*	Presenilin 1
NM_011183	*Psen2*	Presenilin 2
NM_008458	*Serpina3c*	Serine (or cysteine) peptidase inhibitor, clade A, member 3C
NM_009221	*Snca*	Synuclein, alpha
NM_033610	*Sncb*	Synuclein, beta
NM_026842	*Ubqln1*	Ubiquilin 1
NM_025407	*Uqcrc1*	Ubiquinol-cytochrome c reductase core protein 1
NM_025899	*Uqcrc2*	Ubiquinol cytochrome c reductase core protein 2

**Figure 2:**
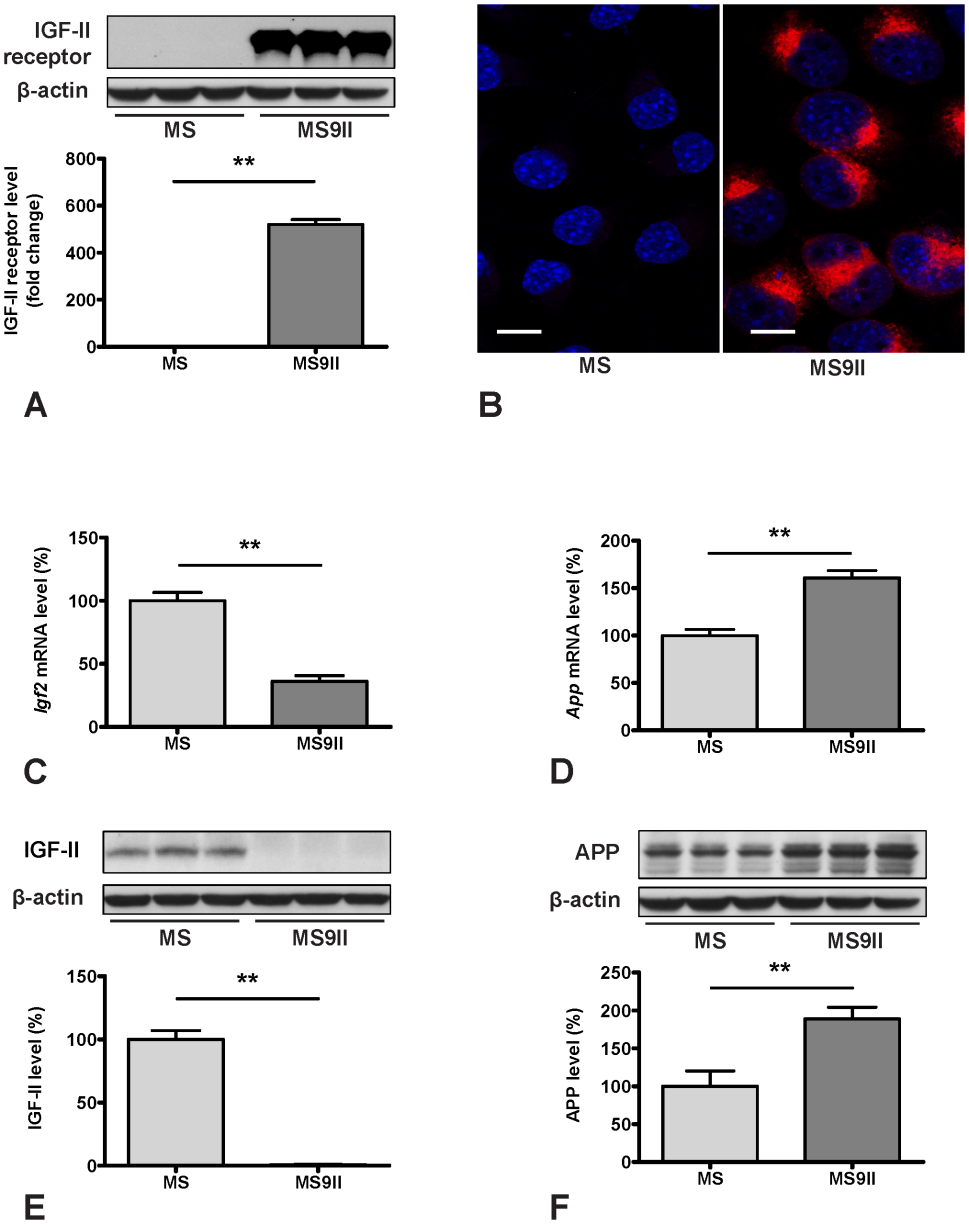
Transcript and protein expression levels of IGF-II receptor (A, B), IGF-II (C, E) and APP (D, F) in MS and MS9II cells. Increased levels and expression of IGF-II receptor in MS9II *vs* MS cells are validated using Western blotting and immunofluorescence staining respectively (A, B). Histograms showing decreased level of *Igf2* mRNA (C) and increased level of *App* mRNA in MS9II cells compared to MS cells as obtained using AD-PCR-Array. Immunoblots and respective histograms validating decreased levels of IGF-II and increased levels of APP in MS9II cells. The protein levels were normalized to the β-actin and the values from four different experiment are expressed as means ± SEM, **p<0.01. Scale bar = 10 µm.

**Figure 3:**
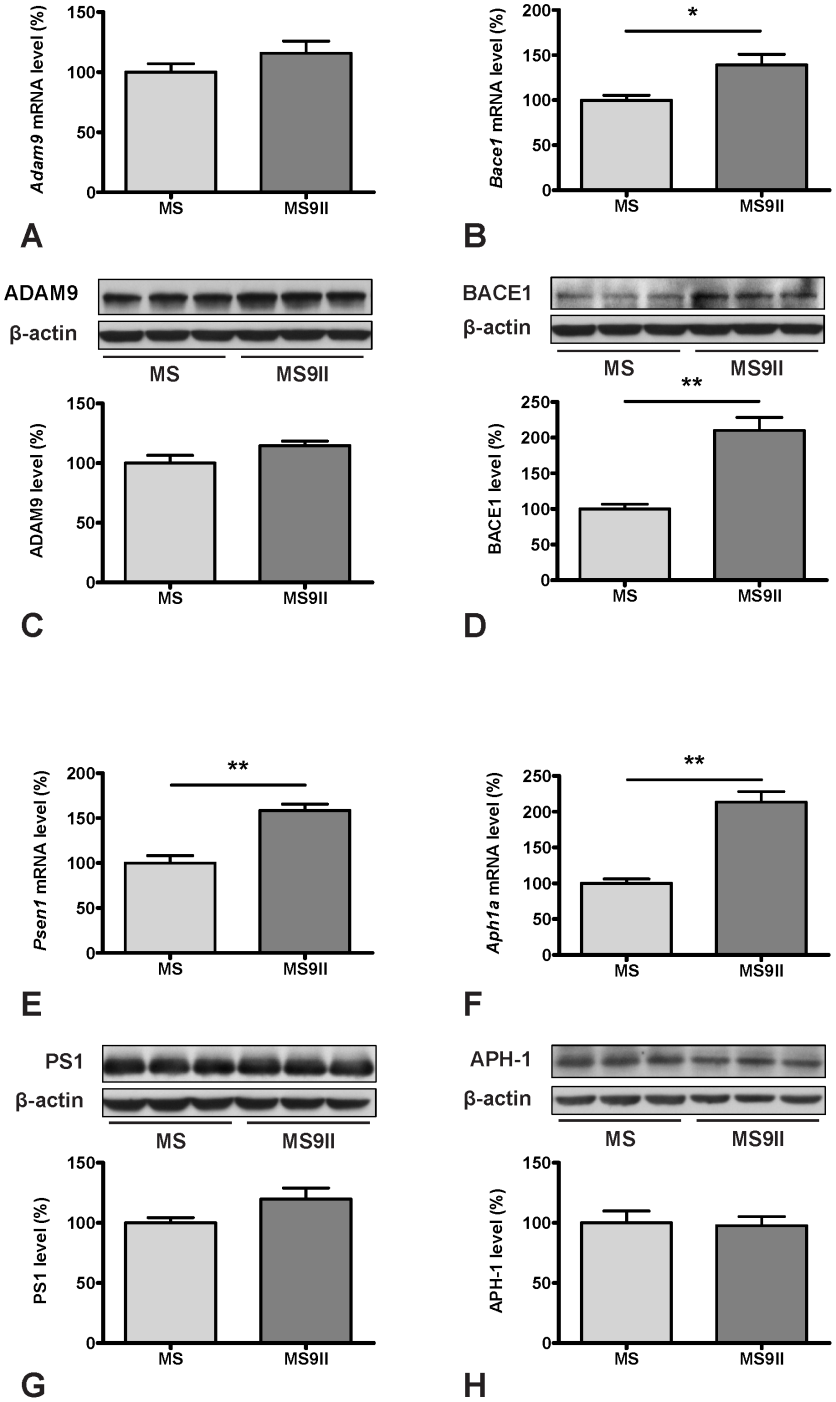
Transcript and protein expression levels of ADAM9 (A, C), BACE1 (B, D), PS1 (E, G) and APH-1 (F, H) in MS and MS9II cells. Histograms showing unaltered levels of *Adam9* mRNA (A) and increased levels of *Bace1* mRNA (B) in MS9II cells compared to MS cells. Immunoblots and respective histograms validating unchanged ADAM9 (C) and increased BACE1 (D) levels in MS9II cells. Histograms showing increased mRNA levels for *Psen1* (E) and *Aph1a* (F) in MS9II cells compared to MS cells. Immunoblots and respective histograms showing unaltered protein levels of PS1 (G) and APH-1 (H) in MS9II cells. The protein levels were normalized to the β-actin and the values from four different experiments are expressed as means ± SEM, *p<0.05, **p<0.01.

**Figure 4:**
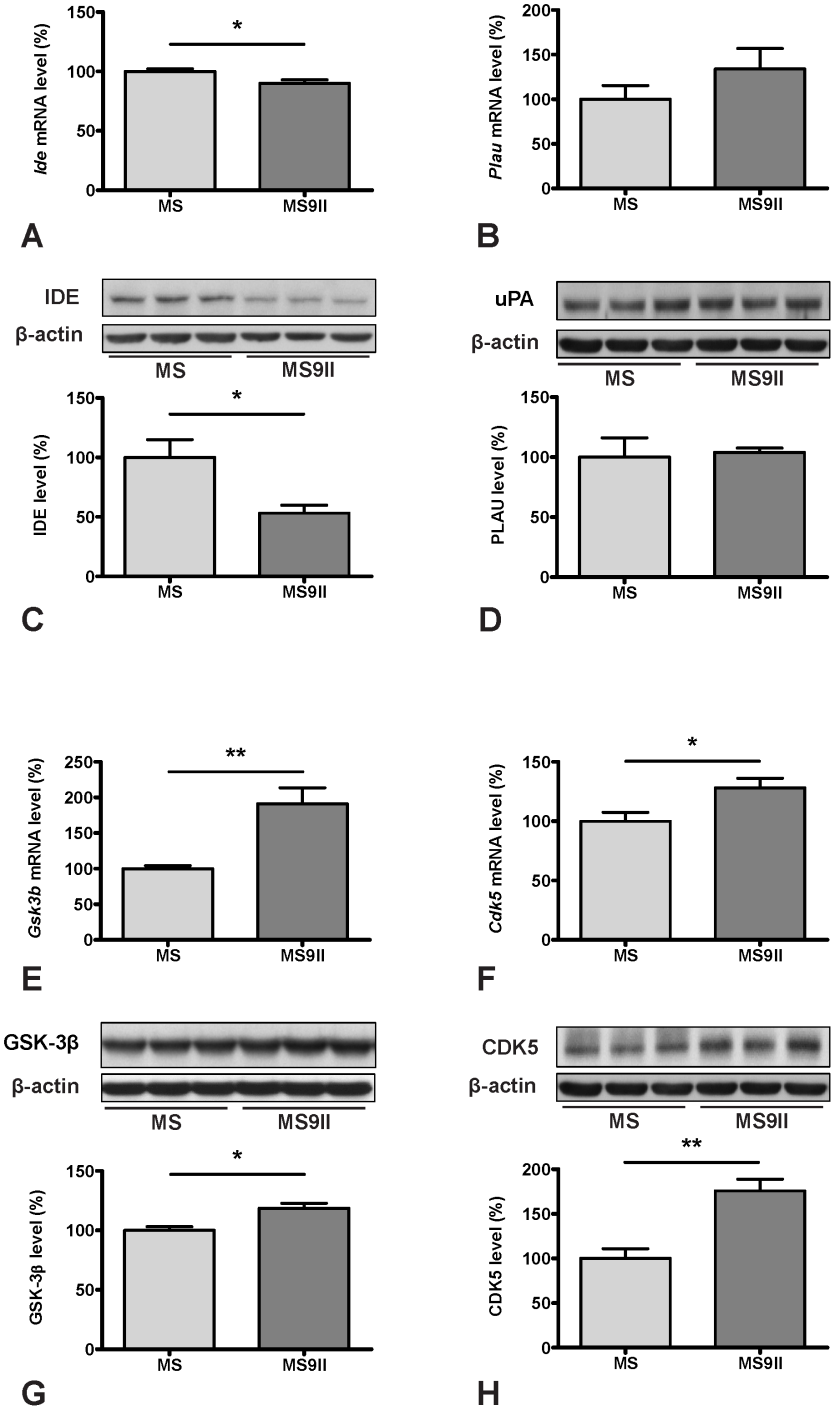
Transcript and protein expression levels of IDE (A, C), PLAU (B, D), GSK-3β (E, G) and CDK5 (F, H) in MS and MS9II cells. Histograms showing decreased levels of *Ide* mRNA (A) and unaltered levels of *Plau* mRNA (B) in MS9II cells compared to MS cells. Immunoblots and respective histograms validating decreased IDE (C) and unchanged uPA (D) levels in MS9II cells. Histograms showing increased mRNA levels for *Gsk3b* (E) and *Cdk5* (F) in MS9II cells compared to MS cells. Immunoblots and respective histograms showing marked increase in protein levels of GSK-3β (G) and CDK5 (H) in MS9II cells. The protein levels were normalized to the β-actin and the values from four different experiments are expressed as means ± SEM, *p<0.05, **p<0.01.

**Figure 5:**
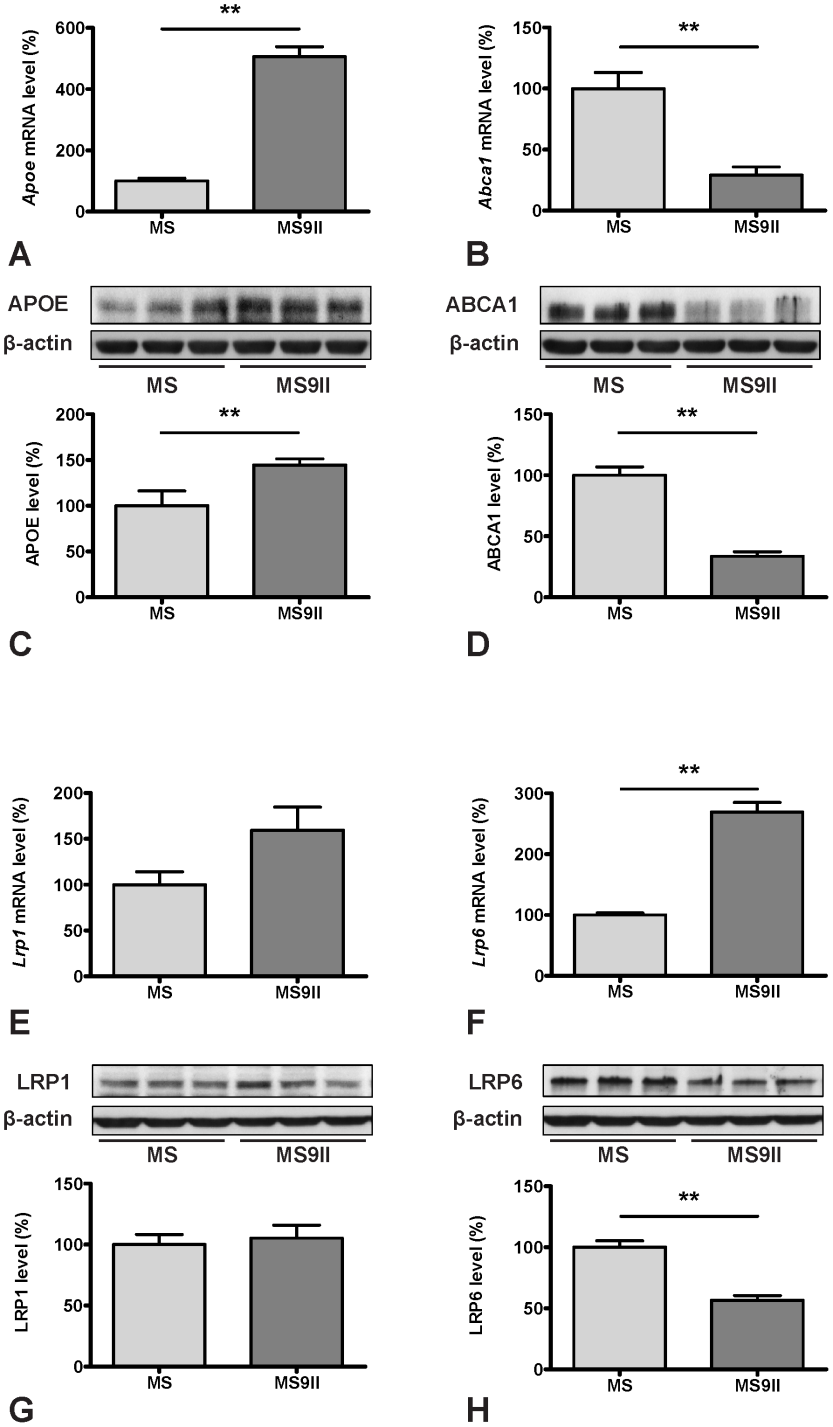
Transcript and protein expression levels of APOE (A, C), ABCA1 (B, D), LRP1 (E, G) and LRP6 (F, H) in MS and MS9II cells. Histograms showing increased levels of *Apoe* mRNA (A) and decreased levels of *Abca1* mRNA (B) in MS9II cells compared to MS cells. Immunoblots and respective histograms validating increased APOE (C) and decreased ABCA1 (D) levels in MS9II cells. Histograms showing unaltered levels of *Lrp1* mRNA (E) and increased levels of *Lrp6* mRNA (F) in MS9II cells compared to MS cells. Immunoblots and respective histograms showing unchanged LRP1 but decreased levels of LRP6 in MS9II cells. The protein levels were normalized to the β-actin and the values from four different experiments are expressed as means ± SEM, *p<0.05, **p<0.01.

**Figure 6:**
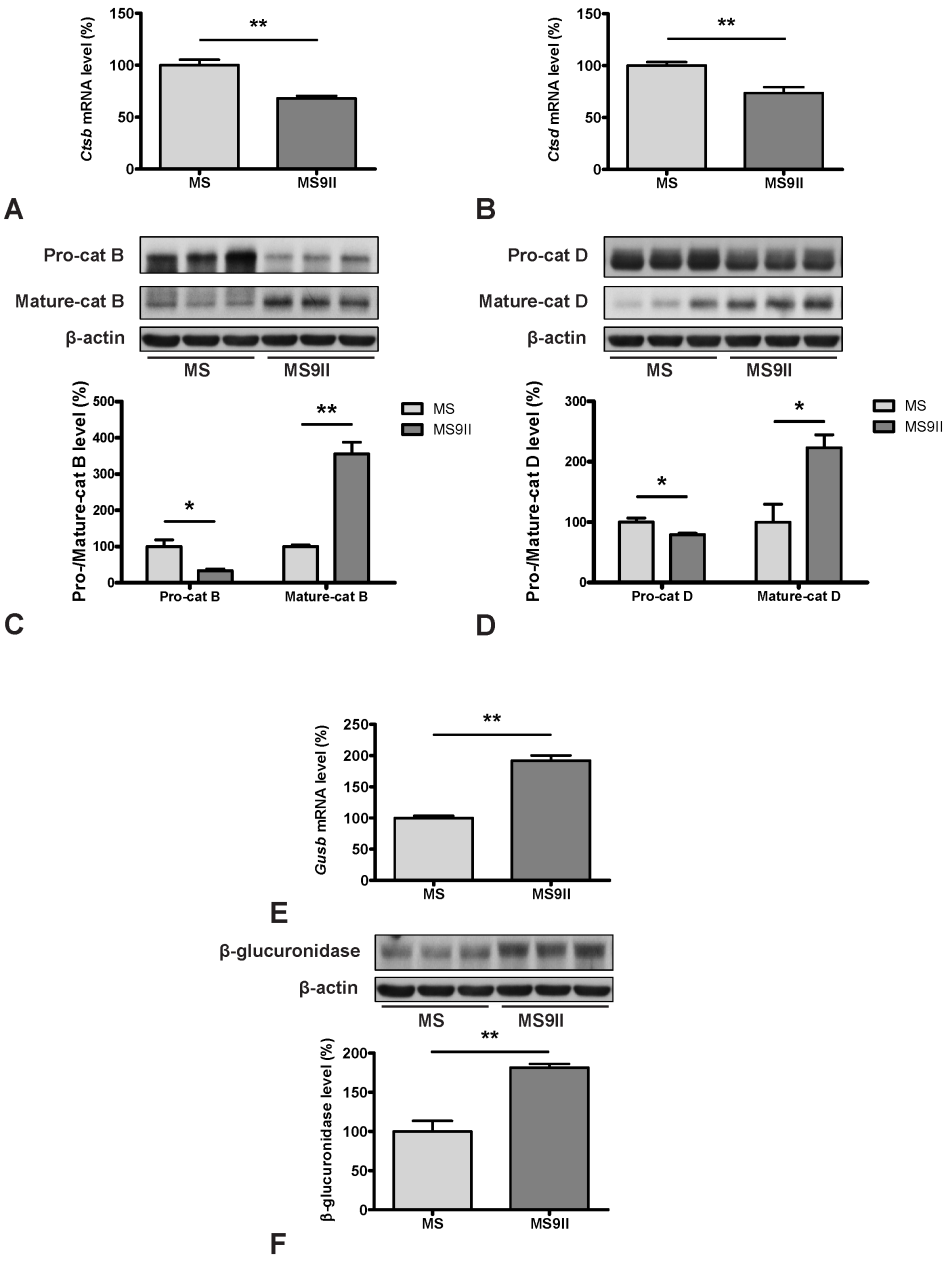
Transcript and protein expression levels of cathepsin B (A, C), cathepsin D (B, D) and β-glucorinidase (E, F) in MS and MS9II cells. Histograms showing decreased levels of C*tsb* mRNA (A) and *Ctsd* mRNA (B), but increased levels of *Gusb* mRNA (E) in MS9II cells compared to MS cells. Immunoblots and respective histograms showed decreased levels of pro-cathepsin B and D but increased levels of mature cathepsins B and D in MS9II cells than in MS cells. Immunoblot analysis of β-glucuronidase level, consistent with mRNA, was enhanced in MS9II cells. The protein levels were normalized to the β-actin and the values from four different experiments are expressed as means ± SEM, *p<0.05, **p<0.01.

AD-PCR-Array data revealed that expression of genes directly involved in Aβ production such as *App*, *Bace1*, *Psen1, Ncstn* and *Aph1a* but not *Adam9* were significantly increased in MS9II cells compared to MS cells (Figs. 1A; 2D; 3A, B, E and F). In contrast, expression of some of the genes involved in Aβ degradation such as *Ide* was decreased, while the others such as *A2m* and *Plau* encoding urokinase-type plasminogen activator (uPA), which activates an Aβ degrading enzyme plasminogen, remains unaltered (Figs. 1A; 4A and B). A number of transcripts that may have a role in regulating Aβ-mediated toxicity [7], [8] were either increased (such as *GSk3a*, *Gsk3b*, *Prkca*, *Casp3*, *Casp4*, *Cdk5*) or showed no alterations (i.e. *Cdk11*, *Prkcc*) in MS9II *vs* MS cells (Figs. 1A; 4E and F). Given the significance of cholesterol in AD pathology [29]–[32], it is of relevance that some of the transcripts involved in cholesterol metabolism were either markedly increased (i.e. *Apoe, Clu* and *Lrp6*), decreased (i.e. *Abca1*) or remained unaltered (i.e. *Apoa1*, *Lpl*, *Lrp1* and *Lrp8*) in MS9II cells compared to MS cells (Figs. 1A; 5A, B, E and F). Transcripts corresponding to various lysosomal enzymes that are transported by the IGF-II receptor and are known to be involved in AD pathology [15], [16], [33]–[35] were also found to be differentially expressed. While the expression of C*tsb* and C*tsd* were decreased and *Gusb* was increased (Figs. 1A; 6A, B and E), *Ctsg* and *Ctsl* did not exhibit any significant alterations between the two cell lines. Apart from its well established trafficking role, IGF-II receptor is known to mediate certain biological effects of IGF-II by triggering specific cellular signalling pathways [36]–[40]. In fact some of these effects including regulation of acetylcholine release from adult rat brain [39], [40] as well as hypertrophy of myocardial cells [41] are known to be mediated by G protein linked protein kinase C (PKC) pathways. In keeping with these results, we observed that various subunits/isoforms of G proteins as well as PKC were differentially expressed in MS9II cells compared to MS cells. Some of the transcripts such as *Gnao1*, *Gnb1*, *Gnb2*, *Gnb4*, *Gnb5*, *Gng5*, *Gng8*, *Gngt2*, *Prkca*, *Prkcb*, *Prkcd*, *Prkce*, *Prkci* and *Prkcz* were found to be significantly increased while others (i.e. *Gnaz*, *Gng3*, *Gng4*, *Gngt1* and *Prkcc*) did not exhibit any marked changes following overexpression of IGF-II receptor (Fig. 1A).

### Validation of Altered Gene Expression Profiles by Western Blotting

To validate the changes observed in the expression profiles of the genes involved in AD pathology, we evaluated steady-state protein levels of selected transcripts in MS and MS9II cells by immunoblot analysis. Consistent with our AD-PCR-Array data, we observed significant increase in the levels of APP (Fig. 2F) and its processing enzyme BACE1 (Fig. 3D), while the levels of ADAM9 remained unaltered in MS9II cells as compared with MS cells (Fig. 3C). Interestingly, the levels of certain proteins with long half-lives such as PS1 and APH1, in contrast to their respective transcripts, were found to be unaltered, consistent with the reports that stable formation of γ-secreatse complex determines the steady-state protein levels of these proteins (Figs. 3G and H) [42]–[44]. In keeping with the transcript levels, we observed significant decrease in the levels of IDE and ABCA1 (Figs. 4C; 5D), marked increase in GSK3β, CDK5 and APOE levels (Figs. 4G and H; 5C) and no alteration in uPA and LRP1 levels (Figs. 4D; 5G) in MS9II cells *vs* MS cells. The levels of LRP6, in contrast to its transcript, were found to be decreased (Fig. 5H) suggesting post-translational regulatory mechanism likely contributes to the steady-state protein levels. With regards to the lysosomal enzymes, the pro-forms of cathepsins B and D correlated rather well with the decreased levels of both transcripts observed in MS9II cells compared to MS cells (Figs. 6C and D). However, the mature forms of these enzymes were found to be markedly increased suggesting an intriguing post-translation mechanism that may have a role in regulating the levels/activity of these enzymes within the cells (Figs. 6C and D). On the other hand, both the *Gusb* transcript levels and the steady-state β-glucuronidase protein levels were higher in MS9II cells as compared with MS cells (Fig. 6F).

## Discussion

The present study using real-time RT-PCR arrays reveals that an increase in IGF-II receptor levels can influence the expression profiles of several genes involved in AD pathology. Notably, some of the differentially expressed genes are directly associated with the production and clearance of Aβ peptides, while the others are linked to cholesterol metabolism and the endosomal-lysosomal system function, all of which are known to play critical roles in the development of AD pathology. The altered gene profiles with few exceptions correlated well with alterations in the corresponding steady-state protein levels. With reference to the quantification of differential gene expression, it is important to highlight two points in context of the results obtained in the present study. First, the absolute fold-changes in the level of a specific transcript need not be of high magnitude to have a significant effect on cell physiology. Second, post-translational modifications on proteins, apart from altered levels of the transcripts, can have an important role in regulating the function and/or development of AD-related pathology. It is also of interest to note that the present study, in the absence of any neuronal cell lines overexpressing IGF-II receptor, was carried out using mouse fibroblast MS9II cells. Thus, the results obtained in the present study may not precisely recapitulate the changes that can be seen in neurons following overexpression of the IGF-II receptor. Nevertheless, these results suggest that an alteration in IGF-II receptor levels can influence the expression profiles of a number of transcripts as well as proteins that are involved either directly and/or indirectly in the development of AD pathology.

### IGF-II Receptor Overexpression and APP/Aβ Metabolism

Previous studies have shown that IGF-II receptor level and expression are increased in the hippocampus and cortex but not in the striatum of mutant APP transgenic mice compared to age-matched control mice [19]. However, the levels/expression of the receptor, unlike transgenic mice, are usually unaltered in AD brains [17], [18], but there is evidence that the receptor levels/expression can be decreased selectively in the hippocampus of AD patients carrying two copies of *APOE* ε4 allele [17] or increased in the cortical region of familial cases with a *PSEN1* mutation [18]. More recently, a quantitative proteomics study reveals that the IGF-II receptor may be a substrate for BACE1, *albeit* its functional significance in relation to AD pathology remains unclear [20]. Our results clearly indicate that overexpression of the IGF-II receptor markedly increases the expression of *App*, *Bace1*, *Psen1, Ncstn* and *Aph1a* but not *Adam9*. Consistent with transcript levels, we observe up-regulation of APP and BACE1 and no alteration in ADAM9 between MS9II and MS cells. The protein levels of PS1 and APH1, unlike their transcripts, are not markedly altered; this is not surprising because it is known that their steady-state levels are tightly regulated by stoichiometric interaction between the four γ-secretase complex subunits [42]–[44]. Nevertheless, it remains to be determined whether IGF-II receptor overexpression can influence γ-secretase enzyme activity. Interestingly, the levels of *Ide* and its corresponding protein insulin degrading enzyme, which is involved in the clearance of Aβ peptides [45], [46], are significantly down-regulated in MS9II cells compared to MS cells. These results suggest that IGF-II receptor overexpression may influence the clearance of Aβ peptides. However, we did not observe an alteration in the expression of *A2m* or *Plau*, which code for two major proteins that mediate Aβ degradation [45]–[47]. Thus it remains to be established to what extent IGF-II receptor can influence Aβ clearance mechanisms, which are known to play an important role in pathogenesis of sporadic AD.

### IGF-II Receptor Overexpression and Cholesterol Metabolism

A number of studies have indicated that altered cholesterol homeostasis can influence AD pathology. This is supported by the evidence that i) inheritance of ε4 isoform of the cholesterol transporter APOE is a major risk factor for late-onset AD [2], [4], ii) epidemiological data suggest statins, drugs that block cholesterol biosynthesis, reduce the prevalence of AD, *albeit* more recent prospective studies have produced conflicting results [48]–[50], iii) elevated cholesterol levels increase Aβ production/deposition, whereas inhibition of cholesterol synthesis lowers Aβ levels/deposition [29], [51]–[53], and iv) some genes related to cholesterol metabolism have been linked to AD including *Clu* (involved in the transport of cholesterol), *LRP* (a major receptor for ApoE in the brain) and *ABCA1* (involved in the efflux of cholesterol), though their associations need to be validated in future studies [54]–[56]. Our real-time RT-PCR array data reflect a dysregulation in cholesterol metabolism following elevation of IGF-II receptor levels as we observed an up-regulation of *Apoe, Clu* and *Lrp6* and down-regulation of *Abca1* in MS9II cells compared to MS cells. Interestingly, *Apoa1*, *Lpl*, *Lrp1* and *Lrp8* transcripts did not exhibit marked alterations between the two cell lines. Consistent with the transcripts levels, we observed an increase in APOE, a decrease in ABCA1 and no alteration in LRP1 levels. The levels of LRP6, in contrast to its transcript, is decreased in MS9II cells than MS cells. Some recent data indicate that APOE can influence AD not only by regulating the transport of cholesterol but also the extent of Aβ fibrilization as well as clearance of Aβ peptides [57], [58]. The ABCA1, on the other hand, has been shown to modulate Aβ deposition by regulating its production as well as lipidation of APOE [59]. Thus, it is possible that IGF-II receptor can influence AD pathology by APOE and ABCA1 regulated cholesterol metabolism.

### IGF-II Receptor Overexpression and Lysosomal Enzymes

IGF-II receptor plays an important role in the transport of newly synthesized lysosomal enzymes from TGN to lysosomes where they regulate the clearance of various cellular proteins [15], [16], [60], [61]. Some of the enzymes such as cathepsins B and D are also known to affect cell viability following their release into the cytosol [28], [60]–[63]. Evidence suggests that cathepsins may be involved in the generation of Aβ peptides and their levels/expressions are increased in the vulnerable neurons as well as plasma of AD patients [33]–[35], [64]–[67]. Inhibitors of cathepsin B or deletion of the gene have been shown to reduce Aβ burden in mutant APP transgenic mice [68], [69]. Interestingly, overexpression of the IGF-II receptor shows a decreased expression of *Ctsb* and *Ctsd* transcripts and pro-forms of the enzymes, while the levels of mature enzymes are increased possibly due to efficient M6P-dependent trafficking and proteolytic conversion of the pro-forms to active enzymes in endosomes and lysosomes [70]. The profile of *Gusb* transcript and the corresponding protein levels, however, are increased following overexpression of the IGF-II receptor levels. Although these results suggest that IGF-II receptor may differentially regulate various lysosomal enzymes, increased levels of mature cathepsins B and D as well as β-glucuronidase, apart from degradation of cellular proteins, can influence AD pathology *via* other pathways including APP/Aβ metabolism.

### IGF-II Receptor Overexpression and Cell Signalling

In contrast to the well established trafficking role of the IGF-II receptor, its significance in triggering intracellular signalling in response to IGF-II binding remains controversial. A number of studies, however, indicate that IGF-II receptor can mediate certain biological effects of IGF-II in multiple cell types, including stimulation of calcium influx in Balb/c-3T3 fibroblasts and CHO cells [36], motility of human rhabdomyosarcoma cells [37], migration of human extravillous trophoblasts [71], stimulation of Na^+^/H^+^ exchange and inositol trisphosphate production [38] and insulin exocytosis by pancreatic β cells [72]. Some of these ligand-induced responses of the IGF-II receptor are triggered by interaction with G protein-induced PKC-dependent signalling pathways [14], [73]. In addition, we have earlier reported that IGF-II receptor activation can potentiate acetylcholine release *via* a G protein sensitive PKCα-dependent pathway in the brain [39], [40]. The results of the present study show that IGF-II receptor overexpression can induce marked alterations in the levels of various transcripts associated with G protein subunits and PKC isoforms. Although significance of these alterations remains to be established, it is possible that these changes may partly be linked to the signalling effects of the IGF-II receptor. Recently it has been reported that IGF-II, by activating its own receptor, can induce long-term potentiation and promote memory consolidation [74]. This is indeed relevant as IGF-II mRNA levels are decreased in AD brains with the progression of disease pathology [75]. The current study also showed a decrease in IGF-II transcript and protein levels in MS9II cells thus suggesting that overexpression of IGF-II receptor may have role not only in APP/Aβ metabolism but also in regulating AD-related cognitive functions. Additionally, we observed that enhanced expression of the IGF-II receptor can increase transcripts and protein levels of GSK and Cdk5 both of which are associated with toxicity induced by Aβ peptides [1], [7], [8]. Thus, it would be of interest to define whether overexpression of the receptor can render the cells more vulnerable to Aβ-mediated toxicity.

### Conclusion

The present study reports that elevation of IGF-II receptor expression differentially alters not only the expression profiles of various transcripts including APP as well as enzymes regulating Aβ production/clearance mechanisms but also certain lysosomal enzymes and protein kinases that are involved in Aβ metabolism, clearance and toxicity. The overexpression of the IGF-II receptor is also found to alter various genes that regulate cholesterol metabolism, which may be of relevance to AD pathogenesis. Additionally, we observe profound changes in a number of G protein and PKC transcripts, which may be associated with IGF-II receptor signalling. The altered gene profiles, with some exceptions, match with the corresponding protein levels. Collectively these results suggest that elevation of IGF-II receptor levels can differentially influence the transcription and protein levels of genes that are involved either directly or indirectly with pathogenesis of AD.

## Supporting Information

Table S1 Gene expression profiles in MS9II cell compared to MS cells as studied using real-time RT-PCR arrays.
(DOCX)
