# Supplementary material for: Overexpression of the IGF-II/M6P Receptor in Mouse Fibroblast Cell Lines Differentially Alters Expression Profiles of Genes Involved in Alzheimer’s Disease-Related Pathology
Source: PLoS One. 2014 May 20;9(5):e98057. doi: 10.1371/journal.pone.0098057 (PMC4028253; doi:10.1371/journal.pone.0098057)
Supplement: Table S1 — Gene expression profiles in MS9II cell compared to MS cells as studied using real-time RT-PCR arrays. (DOCX) [file pone.0098057.s001.docx]

Supplementary Table S1. Gene expression profiles in MS9II cell compared to MS cells as studied using real-time RT-PCR arrays.

| Symbol | Fold Change | T-TEST | Fold Up- or Down-Regulation |
| --- | --- | --- | --- |
|  | MS9II vs MS | *p* value | MS9II vs MS |
| *A2m* | 1.23 | 0.328225 | 1.23 |
| *Abca1* | 0.27 | 0.003017 | -3.66 |
| *Ache* | 2.57 | 0.001389 | 2.57 |
| *Actb* | 1.04 | 0.142635 | 1.04 |
| *Adam9* | 1.15 | 0.246121 | 1.15 |
| *Apba1* | 1.95 | 0.002322 | 1.95 |
| *Apba3* | 2.15 | 0.000002 | 2.15 |
| *Apbb1* | 2.15 | 0.000265 | 2.15 |
| *Apbb2* | 1.64 | 0.000019 | 1.64 |
| *Aph1a* | 2.13 | 0.001477 | 2.13 |
| *Aplp1* | 3.92 | 0.000021 | 3.92 |
| *Aplp2* | 3.33 | 0.000173 | 3.33 |
| *Apoa1* | 1.23 | 0.328225 | 1.23 |
| *Apoe* | 5.10 | 0.000022 | 5.10 |
| *App* | 1.61 | 0.000872 | 1.61 |
| *Bace1* | 1.38 | 0.024851 | 1.38 |
| *Bace2* | 1.23 | 0.328225 | 1.23 |
| *Bche* | 7.23 | 0.000476 | 7.23 |
| *Bdnf* | 2.69 | 0.000020 | 2.69 |
| *Casp3* | 1.66 | 0.000506 | 1.66 |
| *Casp4* | 2.26 | 0.001034 | 2.26 |
| *Cdc2a* | 1.79 | 0.001575 | 1.79 |
| *Cdk5* | 1.28 | 0.044179 | 1.28 |
| *Cdkl1* | 1.23 | 0.328225 | 1.23 |
| *Chat* | 1.69 | 0.189813 | 1.69 |
| *Clu* | 2.60 | 0.001987 | 2.60 |
| *Ctsb* | 0.68 | 0.001526 | -1.47 |
| *Ctsc* | 1.23 | 0.328225 | 1.23 |
| *Ctsd* | 0.73 | 0.007870 | -1.37 |
| *Ctsg* | 1.23 | 0.328225 | 1.23 |
| *Ctsl* | 1.01 | 0.803884 | 1.01 |
| *Ep300* | 2.49 | 0.000094 | 2.49 |
| *Ern1* | 1.56 | 0.002157 | 1.56 |
| *Gap43* | 1.31 | 0.273512 | 1.31 |
| *Gapdh* | 0.97 | 0.154508 | -1.04 |
| *Gnao1* | 2.88 | 0.000005 | 2.88 |
| *Gnaz* | 1.23 | 0.328225 | 1.23 |
| *Gnb1* | 2.68 | 0.000046 | 2.68 |
| *Gnb2* | 1.77 | 0.005113 | 1.77 |
| *Gnb4* | 1.60 | 0.043815 | 1.60 |
| *Gnb5* | 1.30 | 0.032424 | 1.30 |
| *Gng10* | 2.03 | 0.001996 | 2.03 |
| *Gng11* | 5.10 | 0.000303 | 5.10 |
| *Gng3* | 1.38 | 0.101129 | 1.38 |
| *Gng4* | 1.23 | 0.328225 | 1.23 |
| *Gng5* | 1.24 | 0.042740 | 1.24 |
| *Gng7* | 2.42 | 0.003412 | 2.42 |
| *Gng8* | 1.39 | 0.000367 | 1.39 |
| *Gngt1* | 1.23 | 0.328225 | 1.23 |
| *Gngt2* | 1.95 | 0.001047 | 1.95 |
| *Gsk3a* | 1.69 | 0.000041 | 1.69 |
| *Gsk3b* | 1.87 | 0.007212 | 1.87 |
| *Gusb* | 1.92 | 0.000054 | 1.92 |
| *Hprt1* | 0.61 | 0.001769 | -1.64 |
| *Hsd17b10* | 1.47 | 0.009514 | 1.47 |
| *Hsp90ab1* | 1.31 | 0.022245 | 1.31 |
| *Ide* | 0.90 | 0.033155 | -1.11 |
| *Igf2* | 0.36 | 0.000205 | -2.81 |
| *Il1a* | 0.12 | 0.008342 | -8.48 |
| *Insr* | 1.67 | 0.005174 | 1.67 |
| *Lpl* | 0.91 | 0.280363 | -1.09 |
| *Lrp1* | 1.58 | 0.087049 | 1.58 |
| *Lrp6* | 2.68 | 0.000193 | 2.68 |
| *Lrp8* | 1.17 | 0.116426 | 1.17 |
| *Mapt* | 2.87 | 0.009922 | 2.87 |
| *Mpo* | 14.18 | 0.000219 | 14.18 |
| *Mtap2* | 1.23 | 0.328225 | 1.23 |
| *Nae1* | 1.61 | 0.000005 | 1.61 |
| *Ncstn* | 1.51 | 0.000269 | 1.51 |
| *Pkp4* | 0.92 | 0.384186 | -1.09 |
| *Plat* | 1.71 | 0.017514 | 1.71 |
| *Plau* | 1.32 | 0.268308 | 1.32 |
| *Plg* | 5.69 | 0.002213 | 5.69 |
| *Prkca* | 1.46 | 0.032787 | 1.46 |
| *Prkcb* | 8.02 | 0.000062 | 8.02 |
| *Prkcc* | 1.06 | 0.728603 | 1.06 |
| *Prkcd* | 1.48 | 0.001064 | 1.48 |
| *Prkce* | 1.54 | 0.043340 | 1.54 |
| *Prkci* | 1.44 | 0.000855 | 1.44 |
| *Prkcq* | 1.23 | 0.328225 | 1.23 |
| *Prkcz* | 2.91 | 0.000609 | 2.91 |
| *Psen1* | 1.60 | 0.001595 | 1.60 |
| *Psen2* | 0.79 | 0.001120 | -1.27 |
| *Serpina3c* | 1.23 | 0.328225 | 1.23 |
| *Snca* | 1.23 | 0.328225 | 1.23 |
| *Sncb* | 2.48 | 0.016844 | 2.48 |
| *Ubqln1* | 1.47 | 0.000505 | 1.47 |
| *Uqcrc1* | 1.32 | 0.004900 | 1.32 |
| *Uqcrc2* | 0.83 | 0.010693 | -1.20 |
